# Meralgia paresthetica: Now showing on 3T magnetic resonance neurography

**DOI:** 10.4102/sajr.v23i1.1745

**Published:** 2019-08-21

**Authors:** Raihaan M. Ally, Mark D. Velleman, Farhana E. Suleman

**Affiliations:** 1 Private Practitioner, Pretoria, South Africa; 2 Department of Radiology, University of Pretoria, Pretoria, South Africa

**Keywords:** Meralgia paresthetica, lateral femoral cutaneous nerve, neuropathy, magnetic resonance neurography, fluid-sensitive sequences

## Abstract

Meralgia paresthetica is a neuropathy of the lateral femoral cutaneous nerve. Traditionally, the diagnosis is based on classical symptoms and signs. In cases where there is a diagnostic dilemma, the role of magnetic resonance imaging has been to exclude other causes for the patient’s presentation, as the small extraspinal peripheral nerves were not well visualised at imaging. The development of 3-Tesla magnetic resonance neurography, however, has made pathology of these nerves more conspicuous.

## Introduction

Meralgia paresthetica is a peripheral neuropathy of the lateral femoral cutaneous nerve (LFCN). The diagnosis is based on the classical symptoms and clinical findings of pain, an unpleasant sensation, numbness and paraesthesia on the anterolateral aspect of the thigh.^1^ Occasionally, the condition may mimic other entities, causing clinical confusion and further investigation may be needed to confirm the diagnosis.^2^ Traditionally, the small peripheral nerves have been difficult to assess on magnetic resonance imaging (MRI), but the development of 3-Tesla (3T) magnetic resonance neurography (MRN) now makes the pathology of these nerves more conspicuous.^3,4,5^

## Discussion

**FIGURE 1:**
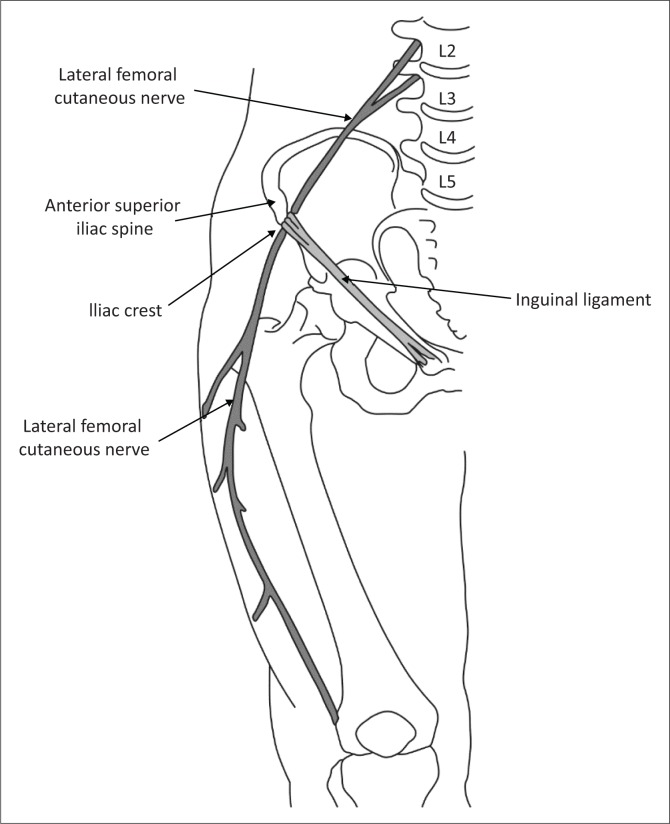
Diagram illustrating the anatomy of the lateral femoral cutaneous nerve.

The LFCN is a purely sensory nerve that may arise from the lumbar nerve roots of L3 alone or in a variable combination of L1, L2 and L3.^4^ It emerges from the lateral aspect of the psoas muscle and crosses the anterior surface of the iliacus muscle across the ilium towards the anterior superior iliac spine (ASIS). It enters the thigh by passing above, below or through the inguinal ligament (Figure 1).^4^

Meralgia paresthetica is a neuropathy of the LFCN that presents with characteristic symptoms and clinical findings. The combined symptoms of pain, numbness and tingling with clinical findings of paraesthesia in the anterolateral thigh is usually typical.^1,4^ The condition may be spontaneous or iatrogenic.^1,6^ Spontaneous development can occur at any age but most commonly occurs in the 30–40-year age group.^1^ There is no consensus about gender predilection in the literature.^4^

Spontaneous causes may be idiopathic, metabolic or due to mechanical compression.^1^ It is most vulnerable to mechanical compression at its most superficial point where it crosses the inguinal ligament.^4^ The wearing of tight clothing, belts and corsets, as well as causes of increased intraabdominal pressure such as pregnancy and obesity, may exert direct pressure on the nerve, resulting in an entrapment neuropathy.^1,2,3,4^ Metabolic causes are associated with diabetes mellitus, alcoholism, lead poisoning and hypothyroidism.^1,2^

Iatrogenic causes include various orthopaedic surgical procedures such as iliac-crest bone graft procedures, anterior pelvic surgery, prone position for spinal surgical procedures and total hip arthroplasty.^1,4^ Anatomical variations of the LFCN occurs in up to 25% of patients and contributes to increased risk of damage during surgery.^1,4^ Five different variations of the course of the LFCN have been described in the literature.^7^ The nerve may run posterior to the ASIS, anterior to the ASIS or medial to the ASIS. These three variations are most vulnerable to injury during surgery.^1^ Further variations include a more medial course of the nerve. Radiation treatment may also be implicated as an iatrogenic cause of lumbosacral neuropathies.^5^

While the diagnosis is based predominantly on history and examination of the patient, the condition may mimic other pathologies of the pelvis and lumbar spine, such as lumbar disc herniation and metastases to the iliac crest.^1^ Investigations used to assist with the diagnosis include needle electrophysiology, nerve conduction studies and electromyography, but all have their limitations.^2,5^ Previously, the role of MRI has been to rule out other pathologies, but subsequent developments in 3T high resolution MRN have allowed better evaluation of pathology of the extraspinal nerves. Thin-slice high resolution fluid-sensitive sequences such as T2 weighted fat saturation (T2FS) or proton density fat saturation (PDFS) are useful. Short Tau Inversion recovery (STIR), diffusion weighted imaging or diffusion-weighted whole-body imaging with background body signal suppression (DWIBS) is also good for identifying signal abnormalities but usually has lower resolution.

**FIGURE 2:**
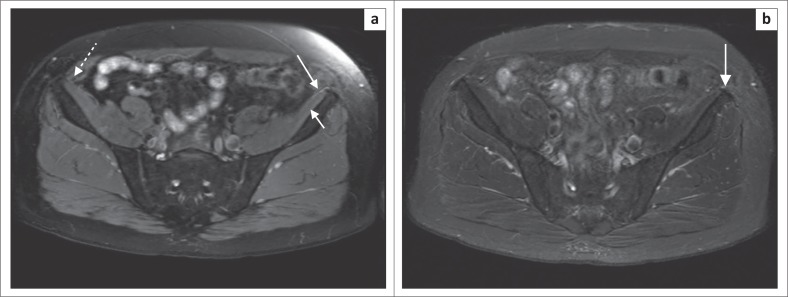
3T proton density fat-saturated axial MR images at the level of the anterior superior iliac spine in a 54-year male patient presenting with a clinical suspicion of meralgia paresthetica on the left. Note the high signal and prominence of the lateral femoral cutaneous nerve on the left (long arrow) as compared to the right (dashed arrow) as it runs over the (a) iliacus muscle (short arrow) and (b) anterior superior iliac spine.

**FIGURE 3:**
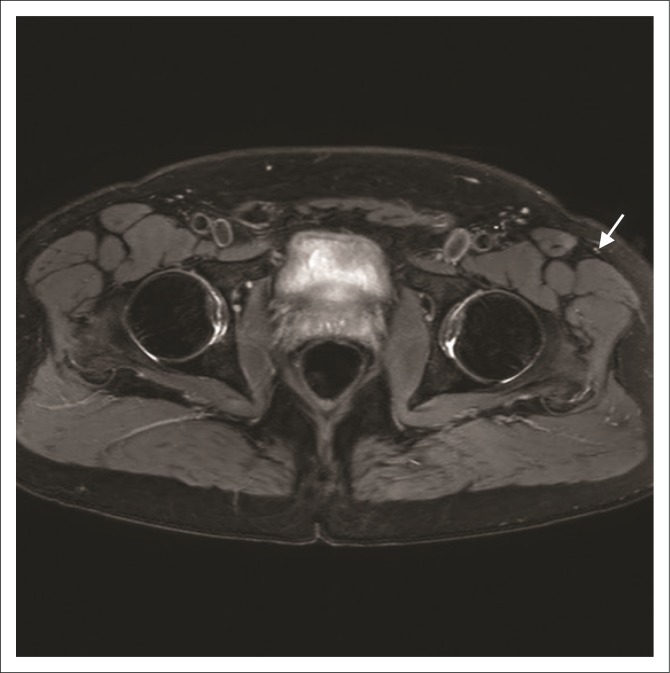
3T proton density fat-saturated axial MR image in the same patient described in Figure 2 demonstrates the persistent high signal of the lateral femoral cutaneous nerve in the subcutaneous tissue where it divides into anterior and lateral branches.

Magnetic resonance neurography allows the detection of changes in perifascicular and endoneurial signal intensity, which may indicate nerve injury. An increase in nerve size, loss of the normal appearance of nerve fascicles and surrounding fat stranding support the findings of nerve injury, but these morphological changes may be more difficult to detect in smaller, more peripheral nerves such as the LFCN.^3^ Motor neuropathies may also be supported by the findings of muscle oedema in a specific nerve distribution, in the acute phase of denervation. This finding will not be present in the purely sensory nerve neuropathy such as in the LFCN in meralgia paresthetica. The diagnosis then depends on findings of signal alteration in the LFCN (Figure 2a and b). It is important to ensure that the skin and subcutaneous tissues are included in the imaging field (Figure 3) to adequately visualise the nerve and its branches.^3^

A study published by Chhabra et al in 2013 investigated the diagnostic accuracy and observer performance of 3T MRN in the investigation of meralgia paresthetica. The study found a moderate inter-observer agreement between two readers for detecting signal alterations of the LFCN with a sensitivity and specificity of ≥71% and ≥94% for both readers, respectively. The authors concluded that 3T MRN was reliable and accurate in the diagnosis of meralgia paresthetica.^2^

## Conclusion

Meralgia paresthetica, a neuropathy of the LFCN, may be spontaneous or iatrogenic. The highly variable anatomy puts it at risk of compression, as well as injury during various surgical procedures. The diagnosis has traditionally been based on clinical findings, but occasionally a diagnostic dilemma may arise. The development of 3T MRN is now proving useful in assisting with the diagnosis of neuropathies of the smaller peripheral nerves.
